# Comparison of effectiveness and safety of camrelizumab between HBV-related and non-B, non-C hepatocellular carcinoma: A retrospective study in China

**DOI:** 10.3389/fgene.2022.1000448

**Published:** 2022-09-09

**Authors:** Haonan Liu, Xiaobing Qin, Zhiyuan Xu, Meng Wu, Tong Lu, Shuang Zhou, Nan Yao, Suya Liu, Yong Shao, Zhengxiang Han

**Affiliations:** ^1^ Department of Oncology, The Affiliated Hospital of Xuzhou Medical University, Xuzhou, China; ^2^ Department of Emergency, The Affiliated Hospital of Xuzhou Medical University, Xuzhou, China; ^3^ Department of Gastroenterology, The Affiliated Hospital of Xuzhou Medical University, Xuzhou, China; ^4^ Department of General Surgery, The Affiliated Hospital of Xuzhou Medical University, Xuzhou, China

**Keywords:** hepatitis B virus, hepatocellular carcinoma, immunotherapy, camrelizumab, effectiveness, safety

## Abstract

**Purpose:** This study aimed to compare the clinical outcomes of camrelizumab in hepatitis B virus-related hepatocellular carcinoma (HBV–HCC) patients and non-HBV, non-HCV hepatocellular carcinoma (NBNC–HCC) patients in China.

**Materials and methods:** A total of 54 patients with hepatocellular carcinoma who received camrelizumab were included in this retrospective study from January 2019 to December 2021. The patients were assigned to the HBV–HCC group (*n* = 28) and the NBNC–HCC group (*n* = 26). The primary endpoints were overall survival (OS) and progression-free survival (PFS), and the secondary endpoints were the objective response rate (ORR), disease control rate (DCR), and adverse events (AEs). Multivariate analysis using Cox proportional hazard regression was used to identify independent prognostic factors. A nomogram model was subsequently established based on independent prognostic factors.

**Results:** The mean duration of follow-up was 12.7 ± 3.6 months. The median OS was not determined. The median PFS in the HBV–HCC group was significantly longer than that in the NBNC–HCC group (9.2 vs. 6.7 months, *p* = 0.003). The ORR and DCR in the HBV–HCC group were significantly higher than those in the NBNC–HCC group (ORR, 28.6% vs. 7.7%, *p* = 0.048; DCR, 71.4% vs. 42.3%, *p* = 0.031). No significant differences in the total incidence of AEs were found between the HBV–HCC group and the NBNC–HCC group (75.0% vs. 69.2%, *p* = 0.224). Multivariate regression analysis identified etiology, AFP level, and vascular invasion as independent prognostic factors (all *p* < 0.05).

**Conclusion:** Our findings demonstrate that camrelizumab is more effective in HBV–HCC patients than in NBNC–HCC patients, with manageable safety.

## Introduction

Hepatocellular carcinoma (HCC) is the seventh most common malignancy and the third most common cause of cancer death worldwide. Specifically, HCC cases in China comprise approximately more than half of the total number of cases globally (Zheng et al., 2018; Sung et al., 2021; Xia et al., 2022).

Chronic infection with the hepatitis B virus (HBV) and hepatitis C virus (HCV) is a major risk factor for HCC; in China, HCC caused by HBV comprises approximately 70%–80% (Wang et al., 2017). With the recent popularization of the HBV vaccine and the widespread application of antiviral drugs, the proportion of HBV-related hepatocellular carcinoma (HBV–HCC) is expected to gradually decrease, whereas that of non-HBV non-HCV hepatocellular carcinoma (NBNC–HCC) is expected to gradually increase (Thomas, 2019). The pathogenesis of NBNC–HCC is yet to be fully understood, but nonalcoholic steatohepatitis (NASH) and metabolic syndrome are regarded as important pathogenic factors (Anstee et al., 2019).

HCC is typically insidious and mostly diagnosed in the intermediate to advanced stages (Maluccio and Covey, 2012). Less than 30% of those diagnosed with the disease undergo radical resection (Ikeda et al., 2018). Immunotherapy provides an option for patients with advanced HCC (Cabibbo et al., 2010; Yoon et al., 2018). Results of the IMbrave150 study show that atezolizumab combined with bevacizumab (T + A) significantly improved overall survival (OS) and progression-free survival (PFS) (Cheng et al., 2022). In addition, the combination (T + A) was approved for the first-line treatment of advanced HCC (Finn et al., 2020; Cheng et al., 2022). It is the first treatment regimen significantly superior to sorafenib in more than 10 years since the approval of sorafenib for first-line treatment of HCC (Sangro et al., 2021). On the basis of the results of the Checkmate040 and KEYNOTE224 trials, two programmed cell death protein 1 (PD-1) inhibitors—nivolumab and pembrolizumab—were approved by the United States Food and Drug Administration (FDA) for second-line therapy of HCC (El-Khoueiry et al., 2017; Zhu et al., 2018). Qin et al. (2020) found that camrelizumab could yield an objective response rate (ORR) of 14.7% and a 6-month OS rate of 74.4% in 217 HCC patients. This finding indicates that camrelizumab could achieve clinical efficacy comparable to that of similar PD-1 inhibitors. Thus, camrelizumab became the first PD-1 inhibitor approved in China for the treatment of intermediate and advanced HCC. In addition, the most significant advantage of camrelizumab for Chinese patients is its relatively low price, hence its wide use in China (Chen et al., 2020).

Although immunotherapy has achieved satisfactory efficacy in HCC patients, international multicenter phase III studies showed that the response rate of NBNC–HCC to immunotherapy was lower than that of viral hepatitis-related HCC (Pfister et al., 2021; Ji and Nguyen, 2022; Kelley et al., 2022). The efficacy of immunotherapy may be influenced by different underlying etiologies of HCC, and different hepatic environments can significantly affect HCC cell induction and immune responses (Roderburg et al., 2020). Compared with non-viral-related HCC, the tumor immune microenvironment of HBV–HCC had a stronger immunosuppressive effect, which was reversed by PD-1 inhibitors (Liu et al., 2021). In addition, viral antigens expressed by tumor cells have been found to increase the number of antigen-specific T cells and enhance response to immunotherapy (Yarchoan et al., 2017).

However, research is lacking on immunotherapy for NBNC–HCC in China. Therefore, in order to identify clinically relevant factors potentially influencing the response to immunotherapy in HCC patients, this study aims to compare the effectiveness and safety of camrelizumab for HBV–HCC and NBNC–HCC in China and to further select HCC patients suitable for immunotherapy by etiology.

## Materials and methods

### Study design and patients

We retrospectively reviewed the medical records of patients with HCC who underwent camrelizumab from January 2019 to December 2021 at the Affiliated Hospital of Xuzhou Medical University.

The inclusion criteria were as follows: (Sung et al., 2021) diagnosed advanced HCC with clinical and histopathological evidence; (Zheng et al., 2018) aged 18 years or older; (Xia et al., 2022) at least one measurable lesion as defined by modified Response Evaluation Criteria in Solid Tumors (mRECIST); (Wang et al., 2017) received at least two cycles of camrelizumab; (Thomas, 2019) Child–Pugh class A or B; (Anstee et al., 2019) Barcelona Clinic Liver Cancer (BCLC) stage B or C; and (Maluccio and Covey, 2012) Eastern Cooperative Oncology Group Performance Status (ECOG PS) ≤ 2.

The exclusion criteria were as follows: (Sung et al., 2021) patients with other malignant tumors; (Zheng et al., 2018) intrahepatic cholangiocarcinoma, and combined hepatocellular–cholangiocarcinoma; (Xia et al., 2022) coinfection with hepatitis C or other hepatitis viruses; and (Wang et al., 2017) incomplete clinical data.

### Grouping and treatment protocol

All included patients were assigned to two groups—the HBV–HCC group and the NBNC–HCC group—on the basis of serum hepatitis B surface antigen, serum hepatitis C surface antigen, HBV–DNA, and HCV–DNA. The HBV–HCC group consisted of patients with positive serum hepatitis B surface antigen or positive serum HBV–DNA, and the NBNC–HCC group consisted of patients with negative serum hepatitis B surface antigen, negative serum hepatitis C surface antigen, negative serum HBV–DNA, and negative serum HCV–DNA.

Camrelizumab (200 mg/branch) was administered intravenously, one branch at a time, once every 3 weeks.

### Assessment

The patients underwent contrast-enhanced chest computed tomography (CT) or contrast-enhanced magnetic resonance imaging (MRI) of the upper abdomen at baseline and every 6–8 weeks thereafter. The long-term efficacy of treatment was measured by OS and PFS. OS was defined as the time from the start of camrelizumab treatment to death from any cause, and PFS was defined as the time from the start of camrelizumab treatment to disease progression or death from any cause. The short-term efficacy was assessed in accordance with the mRECIST criteria, including complete response (CR), partial response (PR), stable disease (SD), and progressive disease (PD). All adverse events (AEs) were recorded in detail, and AEs were evaluated based on the National Cancer Institute Common Terminology Criteria for Adverse Events (version 5.0).

### Statistical analysis

All data were statistically analyzed using the software SPSS 23.0 and R 4.2.0. Normally distributed continuous variables were presented as the mean ± standard deviation and analyzed using the independent *t*-test. Categorical data were expressed as counts and percentages and then analyzed using the chi-squared test or Fisher’s exact probability test. The Kaplan-Meier method was used to estimate median OS and PFS. Log-rank tests were used in univariate analysis, and variables with a *p* value less than 0.1 were used in multivariate analysis. Multivariate Cox proportional hazard regression analysis was used to clarify the independent prognostic factors of PFS. A nomogram prediction model was constructed using the independent prognostic factors from multivariate analysis. Nomograms for 6-month PFS and 9-month PFS were also developed and then compared with the actual scenario. Internal validation and the accuracy of the nomogram were determined using the bootstrap method and the calculated concordance index (C-index). *p* value <0.05 was considered statistically significant.

## Results

### Patient characteristics

**FIGURE 1:**
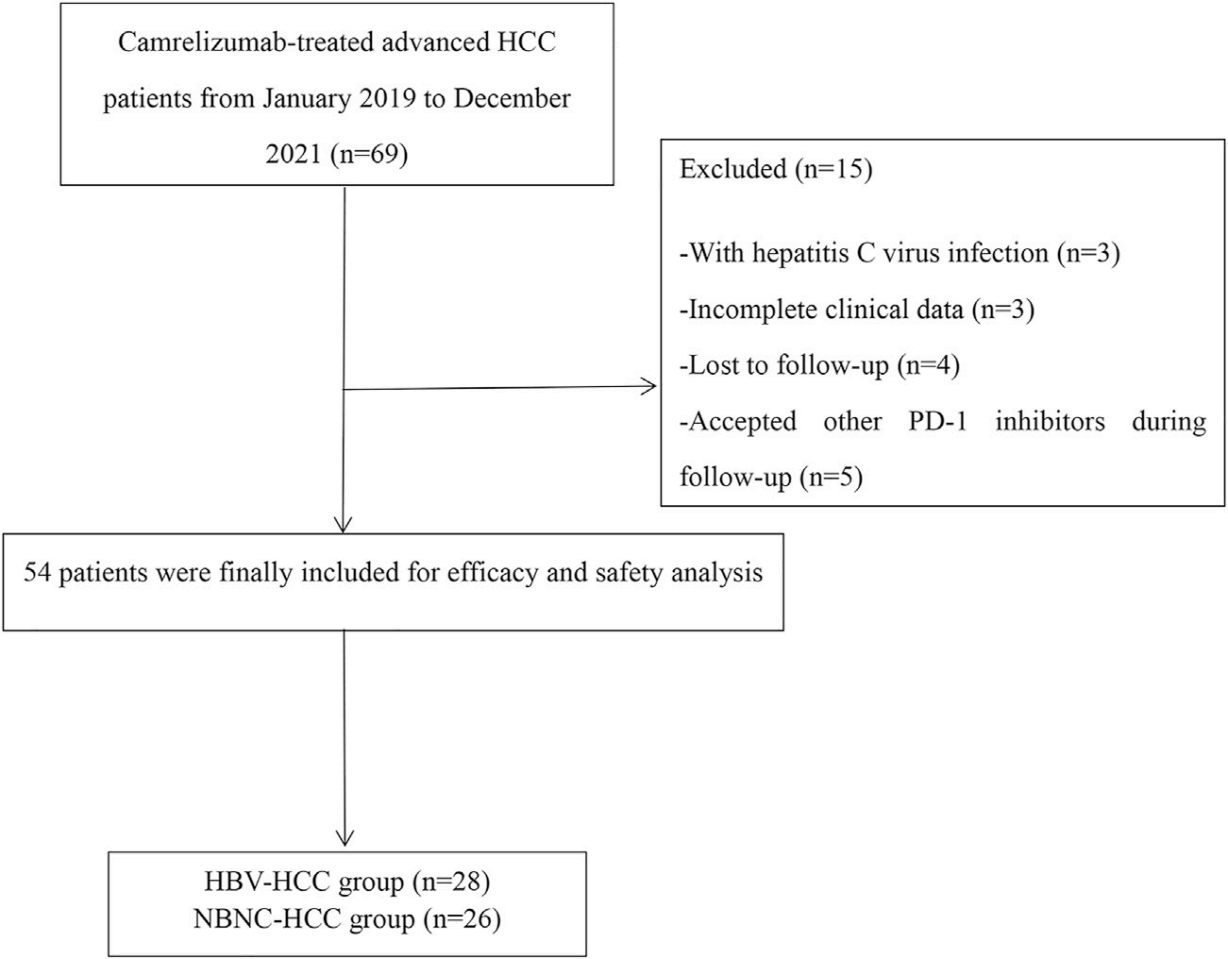
Patient flowchart.

A total of 54 patients with HCC classified as BCLC stage B or C and who are receiving camrelizumab from January 2019 to December 2021 were included in this retrospective study. The HBV–HCC group consisted of 28 patients, and the NBNC–HCC group had 26 patients. The mean follow-up time was 12.7 ± 3.6 months as of March 2022. Fifteen patients were excluded for the following reasons: HCV-infected (*n* = 3), with incomplete clinical data (*n* = 3), lost to follow-up (*n* = 4), and accepted other PD-1 inhibitors during follow-up (*n* = 5) (Figure 1). The causes of NBNC–HCC were NAFLD (non-alcoholic fatty liver disease) (*n* = 22), alcoholic liver disease (*n* = 3), and autoimmune liver diseases (*n* = 1). Patient data, including age, gender, tumor size, cirrhosis, vascular invasion, extrahepatic metastasis, ECOG PS, Child–Pugh class, BCLC stage, previous treatment, and laboratory parameters are summarized in Table 1. No significant differences in baseline characteristics were found between the two groups (all *p* > 0.05).

**TABLE 1:** Baseline characteristics of the HBV–HCC and NBNC–HCC groups.

Variable	HBV–HCC (*n* = 28)	NBNC–HCC (*n* = 26)	t/χ^2^	*p*
Age (years)	55.9 ± 11.5	59.5 ± 10.9	−1.192	0.239
Gender			1.327	0.249
Male	24 (85.7)	19 (73.1)		
Female	4 (14.3)	7 (26.9)		
Tumor size			0.059	0.808
<5 cm	12 (42.9)	12 (30.8)		
≥5 cm	16 (57.1)	14 (53.8)		
Cirrhosis			0.017	0.897
Yes	23(82.1)	21(80.8)		
No	5(17.9)	5(19.2)		
Vascular invasion			0.247	0.619
Yes	10 (35.7)	11 (42.3)		
No	18 (64.3)	15 (57.7)		
Extrahepatic metastasis			0.051	0.821
Yes	11 (39.3)	11 (42.3)		
No	17 (60.7)	15 (57.7)		
ECGO PS			0.044	0.835
0-1	18 (64.3)	16 (61.5)		
2	10 (35.7)	10 (38.5)		
Child-Pugh class			0.027	0.869
A	21 (75.0)	20 (76.9)		
B	7 (25.0)	6 (23.1)		
BCLC stage, *n* (%)			1.169	0.280
B	6 (21.4)	9 (34.6)		
C	22 (78.6)	17 (65.4)		
Previous treatment			0.974	0.968
First-line	9 (32.1)	8 (30.8)		
Second-line	13 (46.4)	10 (38.5)		
Third-line or more	6 (21.4)	8 (30.8)		
Laboratory parameters				
AFP			0.350	0.554
<400 ng/ml	15 (53.6)	16 (61.5)		
≥400 ng/ml	13 (46.4)	10 (38.5)		
AST (U/L)	42.1 ± 13.9	36.9 ± 16.1	1.268	0.210
ALT (U/L)	35.1 ± 20.3	29.0 ± 14.5	1.264	0.212
ALB (g/L)	39.8 ± 5.0	40.9 ± 5.3	−7.490	0.457
TBIL (µmol/L)	18.1 ± 9.0	17.1 ± 8.2	0.460	0.648
PT (s)	12.62 ± 1.45	11.97 ± 1.01	1.907	0.062

Data in brackets represent percentages. HBV–HCC, hepatitis B virus-related hepatocellular carcinoma; NBNC–HCC, non-HBV; non-HCV, hepatocellular carcinoma; ECOG PS, Eastern Cooperative Oncology Group Performance Status; AFP, a-fetoprotein; AST, aspartate aminotransferase; ALT, alanine aminotransferase; TBIL, total bilirubin; PT, prothrombin time.

### Tumor response

In the HBV–HCC group, 1 patient had CR, 7 patients had PR, 12 patients had SD, and 8 patients had PD. In the NBNC–HCC group, no patients showed CR, 2 patients exhibited PR, 9 patients had SD, and 15 patients had PD (Table 2). The ORR and disease control rate (DCR) of the HBV–HCC group were greater than those of the NBNC–HCC group (ORR, 28.6% vs. 7.7%, *p* = 0.048; DCR, 71.4% vs. 42.3%, *p* = 0.031).

**TABLE 2:** Tumor response between the HBV–HCC and NBNC–HCC groups.

Variable	CR	PR	SD	PD	ORR	DCR
HBV–HCC (*n* = 28)	1 (3.6)	7 (25.0)	12 (42.9)	8 (28.6)	8 (28.6)	20 (71.4)
NBNC–HCC (*n* = 26)	0 (0.0)	2 (7.7)	9 (34.6)	15 (57.7)	2 (7.7)	11 (42.3)
χ^2^					3.895	4.676
*P*					0.048	0.031

Data in brackets represent percentages. HBV–HCC, hepatitis B virus-related hepatocellular carcinoma; NBNC–HCC, non-HBV; non-HCV, hepatocellular carcinoma; CR, complete response; PR, partial response; SD, stable disease; PD, progressive disease; ORR, objective response rate; DCR, disease control rate.

### Progression-free survival and overall survival

**FIGURE 2:**
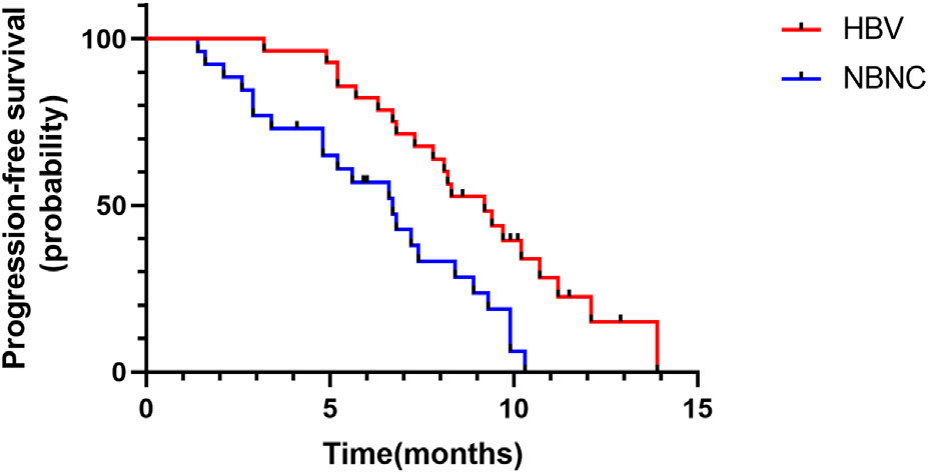
Kaplan–Meier plot of progression-free survival in the HBV–HCC and NBNC–HCC groups.

**FIGURE 3:**
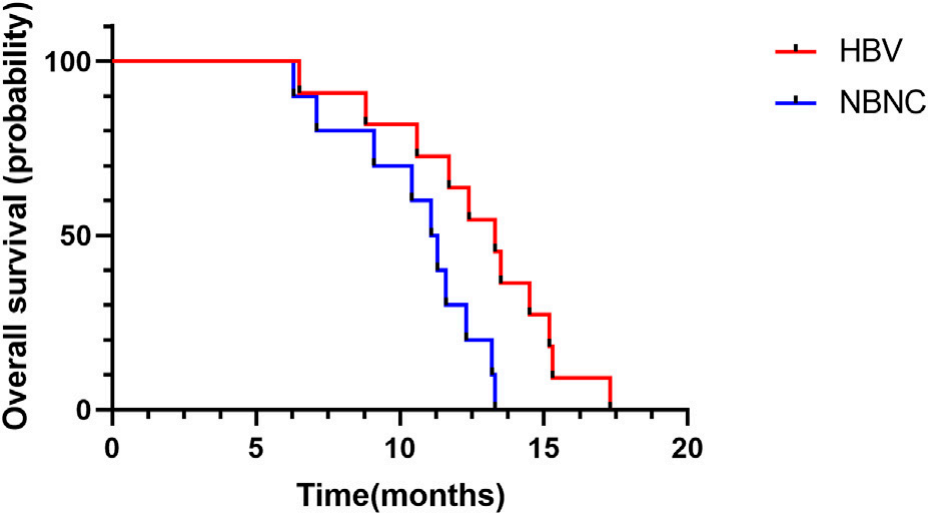
Kaplan–Meier plot of overall survival in the HBV–HCC and NBNC–HCC groups.

Median PFS was 9.2 months (95%CI: 7.4–11.0) in the HBV–HCC group and 6.7 months (95%CI: 5.0–8.4) in the NBNC–HCC group (Figure 2). The median PFS of the HBV–HCC group was significantly longer than that of the NBNC–HCC group (*p* = 0.003). As of follow-up time, 21 patients died, including 11 from the HBV–HCC group and 10 from the NBNC–HCC group. The median OS of the HBV–HCC group was 13.3 months (95%CI: 11.4–15.2), which was significantly longer than that of the NBNC–HCC group (i.e., 11.1 months) (95%CI: 9.7–12.5) (*p* = 0.018) (Figure 3). The median OS still required further observation and follow-up because of the short enrollment time of patients.

### Univariate and multivariate analyses of Progression-free survival

**FIGURE 4:**
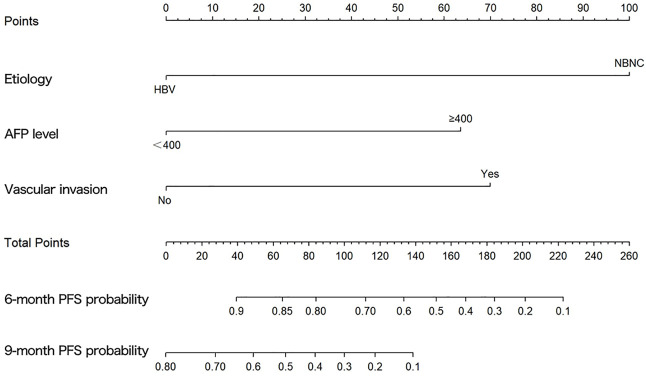
Graph showing the prognostic model for predicting 6- and 9-months progression-free survival.

**FIGURE 5:**
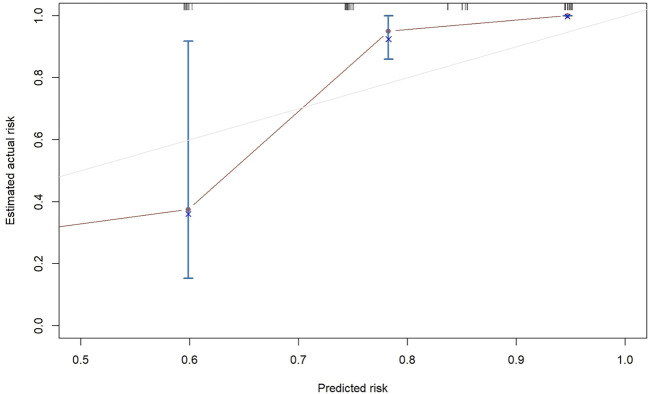
Calibration plots for 6-months progression-free survival.

The results of the univariate and multivariate Cox proportional hazard regression analyses for PFS are summarized in Table 3. In multivariate Cox proportional hazard regression analysis, etiology (HR = 0.192, 95%CI: 0.088–0.418, *p* = 0.001), the AFP level (HR = 2.893, 95%CI: 1.233–6.787, *p* = 0.015), and vascular invasion (HR = 3.158, 95%CI: 1.436–6.942, *p* = 0.004) were identified as independent prognostic factors for the PFS of HCC patients. These independent prognostic factors were considered taken in the construction of a nomogram prediction model (Figure 4). The C-index of the nomogram prediction model was 0.781. The calibration curves indicated consistency between the actual and the predicted survival rates of the patients (Figure 5).

**TABLE 3:** Univariate and multivariate analyses of the prognostic factors for progression-free survival.

Factors	Univariate			Multivariate		
	HR	95% CI	*P*	HR	95% CI	*P*
Age (≥60 vs. < 60), year	1.282	0.693–2.370	0.429	—	—	—
Gender (male vs. female)	0.606	0.275–1.335	0.214	—	—	—
Etiology (HBV vs. NBNC)	0.386	0.202–0.735	0.004	0.192	0.088–0.418	0.001
Tumor size (≥5 vs. < 5), cm	1.304	0.703–2.422	0.400	—	—	—
AFP level (≥400 vs. < 400), ng/ml	2.418	1.236–4.733	0.010	2.893	1.233–6.787	0.015
ECOG PS (0–1 vs. 2)	1.121	0.594–2.115	0.724	—	—	—
Child–Pugh class (A vs. B)	1.183	0.574–2.439	0.648			
BCLC stage (B vs. C)	1.057	0.540–2.070	0.870	—	—	—
Vascular invasion (yes vs. no)	2.889	1.528–5.461	0.001	3.158	1.436–6.942	0.004
Extrahepatic metastasis (yes vs. no)	0.703	0.372–1.326	0.276			
Cirrhosis (yes vs. no)	0.562	0.255–1.241	0.154			

HBV, hepatitis B virus; NBNC, non-HBV, non-HCV; AFP, a-fetoprotein; ECOG PS, eastern cooperative oncology group performance status.

### Adverse events

The two groups of patients mainly reported Grade 1–2 AEs, with reactive cutaneous capillary endothelial proliferation (RCCEP), proteinuria, hyperbilirubinemia, elevated aspartate aminotransferase (AST), elevated alanine aminotransferase (ALT), and thrombocytopenia as the most frequent AEs (Table 4). Grade ≥3 AEs occurred in 13 patients (7 in the HBV–HCC group and 6 in the NBNC–HCC group), and 1 patient in the HBV–HCC group discontinued camrelizumab treatment because of severe AEs (Grade 3 myocarditis). No significant differences in the total incidence of AEs and incidence of grade ≥3 AEs were found between the HBV–HCC group (75.0%, 25.0%) and the NBNC–HCC group (69.2%, 23.1%) (*p* = 0.224, *p* = 0.869).

**TABLE 4:** Treatment-related adverse events in patients with hepatocellular carcinoma.

Effect	All grades		Grade ≥3	
	HBV–HCC (*n* = 28)	NBNC–HCC (*n* = 26)	HBV–HCC (*n* = 28)	NBNC–HCC (*n* = 26)
RCCEP	13 (46.4)	11 (42.3)	0 (0.0)	0 (0.0)
Proteinuria	6 (21.4)	6 (23.1)	0 (0.0)	1 (3.8)
Thrombocytopenia	5 (17.8)	4 (15.4)	0 (0.0)	1 (3.8)
Neutropenia	3 (10.7)	2 (7.7)	0 (0.0)	0 (0.0)
Leukopenia	3 (10.7)	2 (7.7)	0 (0.0)	0 (0.0)
Hypothyroidism	3 (10.7)	4 (15.4)	0 (0.0)	1 (3.8)
Hypertension	4 (14.3)	5 (19.2)	0 (0.0)	1 (3.8)
Rash	2 (7.1)	3 (11.5)	1 (3.6)	0 (0.0)
Nausea	4 (14.3)	3 (11.5)	0 (0.0)	0 (0.0)
Diarrhea	5 (17.9)	4 (15.4)	1 (3.6)	0 (0.0)
Fatigue	4 (14.3)	3 (11.5)	0 (0.0)	0 (0.0)
Myocarditis	1 (7.1)	0 (3.8)	1 (3.6)	0 (0.0)
Hyperbilirubinemia	5 (17.9)	6 (23.1)	1 (3.6)	0 (0.0)
Elevated ALT	7 (25.0)	4 (15.4)	2 (7.1)	1 (3.8)
Elevated AST	6 (21.4)	6 (23.1)	1 (3.6)	1 (3.8)

Data in brackets represent the percentages of patients. HBV–HCC, hepatitis B virus-related hepatocellular carcinoma; NBNC–HCC, non-HBV; non-HCV, hepatocellular carcinoma; RCCEP, reactive cutaneous capillary endothelial proliferation; AST, aspartate aminotransferase; ALT, alanine aminotransferase.

## Discussion

To the best of our knowledge, this research represents the first retrospective study in China that compares the tumor response, survival benefit, and tolerability of camrelizumab treatment for HBV–HCC and NBNC–HCC patients. The results demonstrated that relative to the NBNC–HCC group, the HBV–HCC group showed significant improvement in ORR and DCR. PFS was significantly longer in the HBV–HCC group than in the NBNC–HCC group, and HBV infection was a significant independent predictor of better PFS. In addition, tolerability was similar between the two groups, and no adverse reaction-related deaths occurred.

In 2007, the molecularly targeted drug sorafenib was approved as a first-line treatment for advanced HCC; however, the median survival time was extended by only about 3 months, compared with the placebo (Boland and Wu, 2018). With the understanding of the tumor immune microenvironment and immune targets in recent years, immunotherapy has developed rapidly and achieved ideal efficacy in HCC treatment. Both National Comprehensive Cancer Network (NCCN) Guidelines for Hepatobiliary Cancers and Guidelines for Diagnosis and Treatment of Primary Liver Cancer recommend PD-1/L1 inhibitors as first-line drugs for systemic treatment of HCC (Du et al., 2022; Llovet et al., 2022). Camrelizumab, a PD-1 inhibitor, inhibits the immune escape of liver tumor cells by blocking the binding of PD-1 on CD8 T cells to programmed death ligand-1 (PD-L1) on Kupffer cells (Shen et al., 2019). However, PD-1/L1 inhibitors have exhibited only a 15%–23% response rate in HCC patients (Pinter et al., 2021). HBV infection is the most important cause of HCC; however, baseline HBV loads may not affect the clinical prognosis of PD-1 inhibitor therapy in HCC. Sun et al. (2020) found no significant correlation between clinical outcomes and HBV loads in HCC patients receiving the PD-1 inhibitor. In addition, Yuan et al. (2021) found that HBV loads did not affect the short-term efficacy of the PD-1 inhibitor combined with antiangiogenic therapy for HCC patients.

NAFLD refers to a clinical syndrome characterized by excessive deposition of fat in liver cells, apart from alcohol and other definite liver damage factors (Eslam et al., 2020). Among NAFLD, NASH is considered the most serious; up to 30% of NASH patients may develop liver inflammation and fibrosis, and some patients may further develop to HCC (Stine et al., 2018). NASH activates innate and adaptive immune cells, as well as increases metabolites and endoplasmic reticulum stress, causing hepatic necroinflammation and regenerative cycles, which may lead to HCC (Shen et al., 2017; Xu et al., 2017). Compared with HCC related to viral hepatitis, NBNC–HCC (NAFLD and/or NASH–HCC) is more insidious, and its lesions are mostly single and larger, and the prognosis of patients is worse (Li and Wang, 2022). In addition, several studies have reported that HBV–HCC and NBNC–HCC have significant differences in histopathological characteristics and prognosis (Salomao et al., 2012; Xue et al., 2020). The pathogenesis of NBNC–HCC remains unclear and is currently related to factors such as genetics, metabolism, oxidative stress, immunity, and intestinal flora imbalance (Li and Wang, 2022).

Whether viral infection affects the efficacy of immunotherapy in HCC patients remains inconclusive. In the study by Ho et al. (2020), no noticeable differences in ORR existed between virally infected and uninfected HCC patients receiving PD-1/PD-L1 inhibitors, and viral status could not be a criterion for PD-1/PD-L1 inhibitors. However, recent studies suggest that NBNC–HCC, particularly NASH–HCC, may weakly respond to immunotherapy. Kelley et al. (2022) reported that the benefit of cabozantinib plus atezolizumab treatment to PFS was mainly observed in the HBV–HCC group. Pfister et al. (2021) also found that in a mouse model of NASH, CD8/PD-1 double-positive T cells were unconventionally activated and gradually accumulated in NASH-infected livers (Dudek et al., 2021). Treatment with PD-1 inhibitors increased the number of activated CD8/PD-1 double-positive T cells in NASH–HCC mice but failed to shrink tumors. These results indicate that activated abnormal T cells may not play a role in immune surveillance and cannot kill tumor cells. Thus, Pfister conducted a meta-analysis of three large randomized controlled phase III trials of immunotherapies in patients with advanced HCC (CheckMate-45911, IMbrave1505, and KEYNOTE-24010). The results showed that although immunotherapy improved survival in HBV–HCC patients (HR = 0.64; 95%CI = 0.49–0.83), survival in NBNC–HCC patients was not significantly improved (HR = 0.92; 95%CI = 0.77–1.11) (Pfister et al., 2021). In the current study, similar results were obtained: the median PFS of the HBV–HCC group was significantly longer than that of the NBNC–HCC group (9.2 vs. 6.7 months, *p* = 0.003).

In this study, the incidence of AEs was similar in the two groups—that is, mainly low-grade AEs. One patient in the HBV–HCC group needed to discontinue camrelizumab treatment because of Grade 3 myocarditis; the patient received symptomatic treatment such as hormones and nutritional myocardium. For camrelizumab, RCCEP was the most common AE. The incidence rates of Grade 1–2 RCCEP in the two groups were 46.4% and 42.3%, respectively. No grade ≥3 RCCEP occurred, which was basically consistent with other studies (Qin et al., 2020). The AEs of the two groups were generally controllable, and no fatal AEs occurred.

The results of multivariable Cox proportional hazard regression analysis showed that etiology, AFP level, and vascular invasion were independent prognostic factors for PFS. We then constructed a nomogram prediction model by using these independent risk factors. The nomogram showed that etiology exerted the most effect on PFS, whereas the AFP level exerted the least effect on PFS. To validate the nomogram prediction model, we calculated the C-index and plotted the calibration curve. The C-index of the nomogram model in this study was 0.785, and the calibration curve indicated that our model was consistent with the actual observations, proving the reliability and precision of our model. However, owing to the limited number of cases, no external validation was performed.

This study had several limitations. First, the patients in this study were only treated with camrelizumab, and our conclusions for other PD-1/PD-L1 inhibitors require verification. Second, a stratified analysis of NBNC–HCC etiologies could not be performed because of the small sample size. Third, the median OS for the majority of the population was not determined because of the relatively short follow-up period. We intend to extend the follow-up time in the future. Fourth, only 54 patients were included in the study; the sample size needs to be expanded in the future. Fifth, this study was a single-center retrospective study, and all patients included in this study were from China, where HBV is the main cause of HCC. Finally, some patients had received transarterial chemoembolization and targeted therapy, among others, which might have affected the efficacy of camrelizumab.

## Conclusion

Compared with HBV–HCC patients, NBNC–HCC patients may exhibit a weaker response to PD-1 inhibitors, suggesting that clinicians can screen HCC subgroups suitable for immunotherapy on the basis of etiology to obtain improved efficacy. Large randomized controlled trials are needed in the future to prove our conclusions.

## Data Availability

The original contributions presented in the study are included in the article/Supplementary Material, further inquiries can be directed to the corresponding authors.
